# Differences in Airway Remodeling and Emphysematous Lesions between Rats Exposed to Smoke from New-Type and Conventional Tobacco Varieties

**DOI:** 10.3390/antiox13050511

**Published:** 2024-04-24

**Authors:** Keqiang Wei, Yuanyuan Li, Bin Du, Juan Wu

**Affiliations:** School of Life Science, Shanxi University, Taiyuan 030006, China

**Keywords:** COPD, lung, medicinal plant, oxidative stress, tobacco

## Abstract

Genes from *Perilla frutescens* and *Ocimum basilicum* were introduced into *N. tabacum* L. var. HHY via distant hybridization, and the new-type tobacco varieties “Zisu” and “Luole” were developed, with noticeable differences in chemical composition. Smoking is the leading cause of chronic obstructive pulmonary disease (COPD), and its pathogenesis is complex. In the present study, 48 male Sprague-Dawley (SD) rats were randomly divided into four groups, namely, the control, “HHY”, “Zisu” and “Luole”, and then exposed to fresh air/cigarette smoke (CS) for 30 days and 60 days. The COPD model was constructed, and their health hazards were compared and evaluated. CS from different tobacco varieties influenced rats in varying degrees at the tissue, cell and molecular levels. The rats in the “HHY” group showed obvious symptoms, such as cough and dyspnea, which were less severe in the “Zisu” and “Luole” groups. Pathological and morphological analyses, including scores, MLI, MAN, WAt/Pbm and WAm/Pbm, showed that “Zisu” and “Luole” caused less damage to the airways and lung parenchyma than “HHY”. Significant increases in the numbers of total leukocytes and neutrophils in the BALF were found in “HHY” compared to those in “Zisu” and “Luole”. Moreover, they caused less oxidative stress and apoptosis in lung tissues, as reflected by indicators such as ROS, MDA, T-AOC, GSH, the apoptotic index and the ratio of Bcl-2 to Bax. “Zisu” and “Luole” even altered the ratios of MMP-9/TIMP-1 and IFN-γ/IL-4 in lung tissues to a lesser degree. These differences between CS-exposed rats may be closely related to the altered expression of Nrf2, p38 MAPK and p-p38 MAPK. Changes in chemical composition via introducing genes from some medicinal plants may be an attractive strategy for tobacco harm reduction.

## 1. Introduction

Cigarette smoking is a harmful habit deeply rooted in modern society. Despite continuous efforts to reduce smoking prevalence worldwide, many people still continue to use cigarettes [1]. Cigarette smoke, comprising some 5000 constituents, including free radicals and other oxidants, is considered the leading cause of chronic obstructive pulmonary disease (COPD) [2]. The pathogenesis of COPD is very complex, involving oxidation/antioxidation imbalance, chronic inflammation, cell apoptosis, protease/antiprotease imbalance, etc. Oxidative stress caused by ROS/oxidants, which are contained in cigarette smoke or generated by inflammatory cells, is fundamental in various mechanisms [3,4].

Since the 1950s, people have been trying to make tobacco products less toxic and less harmful. Because many natural active compounds from medicinal or aromatic plants are commonly recognized as effective antioxidants and free radical scavengers, they have been used to eliminate free radicals and reduce harmful components in cigarette smoke by introducing them into the cigarette filter or to manufacture a “less hazardous” “herbal” cigarette by mixing them with or spraying them on tobacco leaves. Some plants and their constituents have been reported for such purposes, including *Ginkgo biloba* extract, *Vitis vinifera* seed extract, *Apocynum venetum* leaves, tea (*Camellia sinensis*) polyphenols and pycnogenol from *Pinus maritima*, as well as lycopene, carotenoids, anthocyanidins, etc. [5,6,7]. Hundreds of natural substances, such as licorice extract and menthol, have been added to cigarettes to change the flavor characteristics of the smoke [5,8]. Some studies also indicated that they do not significantly change the smoke chemistry or the overall toxicity [9,10]. But whether the resulting cigarettes could lessen inherent disease risks or cause new health risks is still highly controversial.

The biological activity of cigarette smoke is determined by its chemical composition, but the tobacco type, or even the variety, is the key determining factor. For example, there are genetic differences among flue-cured (bright), air-cured (burley) and oriental tobacco, leading to their different smoke constituents [9,10,11,12]. Research also showed that the genetic base of modern tobacco cultivars is comparatively narrow, and distant hybridization is an effective measure to create new varieties or new types. In our previous research, new-type tobacco, such as Zisu tobacco and Luole tobacco, was successfully obtained by introducing genes from *P. frutescens* (L.) Britt or *Ocimum basilicum* L. into *N. tabacum* L. var. HHY [13,14,15,16]. Further analysis found that new-type tobacco was quite different from traditional tobacco in the chemical constituents of tobacco leaves, and this resulted in a significant decrease in the in vitro cytotoxic and mutagenic potential of cigarette smoke and greatly lessened lung neutrophilic inflammation in smoking rats [17,18,19]. But the underlying mechanism has not yet been determined.

The lung is especially vulnerable to oxidative insults, and various types of cells release ROS, inflammatory mediators and proteolytic enzymes in response to cigarette smoke, causing airway remodeling (chronic bronchitis) and parenchymal destruction (emphysema) in COPD [3]. At present, there are many difficulties in detecting all components in tobacco smoke, and in vitro toxicity testing of cigarette smoke still has a great limitation in risk assessment [11,20,21,22]. But an in vivo study based on specific cigarette smoke-induced diseases, such as a COPD model, could effectively reflect the biological effects and health risks of tobacco smoke [23,24,25]. Although this method has been recommended for exploring the toxicology and health effects of complex air pollution, so far, there have been no relevant reports on cigarette smoke [26]. Despite its prevalence, the precise mechanisms contributing to COPD are not fully understood, and it appears to be a multifactorial disease [4]. Special attention has been focused on multiple MAPK and Nrf2 signaling-mediated cellular responses and their role in either the pathogenesis or medical intervention of COPD [6]. The objective of this study was to evaluate the changes in oxidative stress, cell apoptosis, Th1/Th2 immune imbalance (i.e., IFN-γ/IL-4) and protease/antiprotease imbalance (i.e., MMP-9/TIMP-1) that are associated with the ROS/MAPK/Nrf2 pathway by following Sprague-Dawley (SD) rats exposed to new-type and conventional tobacco varieties (i.e., “Zisu”, “Luole” and “HHY”).

## 2. Materials and Methods

### 2.1. Tested Cigarettes

The new-type tobacco varieties *N. tabacum* L. var. Zisu and *N. tabacum* L. var. Luole were derived from two cross combinations, namely, [(*N. sylvestris* Speg. & S. Comes + *N. tabacum* L. var. HHY) × *P. frutescens* (L.) Britt var. frutescens] and [(*N. sylvestris* Speg. & S. Comes + *N. tabacum* L. var. HHY) × *O. basilicum* L.], as described previously [14,16]. *N. tabacum* L. var. HHY (pistillate parent) is a sun-cured tobacco variety in China, derived from hybridization between two varieties, viz., “Jintai 33” and “Jiaohe” [27]. *P. frutescens* (L.) Britt var. frutescens and *O. basilicum* L. (pollen parent) are cultivated varieties of medicinal plants in China (Figure S1). “HHY”, “Zisu” and “Luole” were used as test tobacco and planted in experimental plots at Shanxi Agricultural University (Taigu, China). Unfiltered cigarette samples were 57 mm in rod length and 7 mm in diameter and prepared by Shanxi Kunming Tobacco Co., Ltd. (Taiyuan, China). The average tobacco weights in each finished cigarette (n = 20) were 0.709 g, 0.712 g and 0.711 g, respectively. The contents of carbon monoxide (CO), tar and nicotine in cigarettes were determined (mg/cigarette): “HHY”: CO 16.7, tar 17.7, nicotine 1.52 (equivalent to 1.4-, 1.9- and 2.2-fold higher than the quantities in the Kentucky Reference Cigarette 3R4F, respectively); “Zisu”: CO 12.9, tar 11.1, nicotine 1.31 (equivalent to 1.1-, 1.2- and 1.9-fold higher than the quantities in the Kentucky Reference Cigarette 3R4F, respectively); “Luole”: CO 12.6, tar 11.2, nicotine 1.10 (equivalent to 1.1-, 1.2- and 1.6-fold higher than the quantities in the Kentucky Reference Cigarette 3R4F, respectively). All cigarettes were unpacked and conditioned, prior to smoking, in an environmental chamber at a temperature of 22 ± 2 °C and relative humidity of 60 ± 5% for at least 48 h.

### 2.2. Chemicals and Reagents

Rabbit polyclonal antibodies against Bcl-2 (26593-1-AP), Nrf2 (16396-1-AP), p38 MAPK (14064-1-AP) and phospho-p38 MAPK (Thr180/Tyr182) (28796-1-AP) were purchased from Proteintech (Wuhan, China). The anti-Bax rabbit polyclonal antibody (D120073) and Bradford Protein assay kit (C503031) were obtained from Sangon (Shanghai, China). The SABC-POD kit (SA1022) and secondary antibody (Biotin-conjugated goat anti-rabbit IgG, BA1003) were provided by Boster (Wuhan, China). The assay kits for matrix metalloproteinases-9 (MMP-9) (H146-4), Tissue Inhibitor of Metalloproteinases 1 (TIMP-1) (H147-1), Malondialdehyde (MDA) (A003-1), Reduced Glutathione (GSH) (A006-2), Interleukin-4 (IL-4) (H005-1) and Interferon-γ (IFN-γ) (H025-1) were obtained from Nanjing Jiancheng (Nanjing, China). The total antioxidant capacity (T-AOC) assay kit (BC1315) and DAPI solution (C0065) were provided by Solarbio (Beijing, China). DCFH-DA (287810) and the TUNEL BrightGreen Apoptosis Detection Kit (A112) were obtained from Sigma Aldrich (St. Louis, MO, USA) and Vazyme (Nanjing, China), respectively.

### 2.3. Experimental Animals

A total of 48 male SD rats, 6 weeks old with a body weight of 190 ± 10 g, were provided by the Laboratory Animal Center of Shanxi Medical University (Taiyuan, China). They were housed in polypropylene cages (2 rats/cage) and maintained in a 12 h light/dark cycle at a temperature of 23 ± 2 °C and a humidity of 50 ± 5%. The rats were allowed free access to a standard diet and water. After acclimatization for 1 week, they were randomly divided into four groups: the control group, exposed to clean air; group 1, exposed to sidestream smoke (SS) from “HHY” cigarettes; group 2, exposed to SS from “Zisu” cigarettes; and group 3, exposed to SS from “Luole” cigarettes, with twelve rats in each group. The experimental protocols were reviewed and approved by the Committee of Scientific Research of Shanxi University, China (No. SXULL2022090).

### 2.4. Model Establishment and Sampling

A perspex apparatus designed for whole-body exposure to cigarette smoke was prepared as described in the literature [28]. Smoke generated from lighted cigarettes in the burn box was delivered into the exposure chamber (height 60 cm, width 80 cm, length 100 cm) by a circulating fan. The rat COPD model was established according to the protocol of a previous study [28,29] with some modifications. In brief, six conscious and unrestrained rats were exposed to smoke from 12 cigarettes for 60 min at a time, two times a day (at 10:00 a.m. and 3:00 p.m.), 6 days a week for 30 days and 60 days. The control group underwent an identical procedure when inhaling fresh air. The rats were then carefully observed daily.

After 30 days and 60 days of smoke exposure, 6 rats randomly selected in each group were anesthetized by intraperitoneal injection of sodium pentobarbital (40 mg/kg), tracheotomized and cannulated. The left lungs were lavaged 3 times with 1 mL of phosphate-buffered saline (PBS). The bronchoalveolar lavage fluid (BALF) was collected and centrifuged (1500 rpm, 10 min, 4 °C). The middle lobe of the right lung was excised, fixed in 4% paraformaldehyde, embedded in paraffin and subjected to histopathological examination, TUNEL staining and immunohistochemical analysis, respectively. The remaining lung tissue was homogenized with 10% (*w*/*v*) 0.1 M potassium phosphate buffer (pH 7.0) and centrifuged (3000 rpm, 10 min, 4 °C), and the supernatants were further analyzed.

### 2.5. Total and Differential Cell Counts

As described previously [30], the BALF was centrifuged, and the cell pellets were resuspended in 100 µL of PBS. The number of total cells was determined by a hemacytometer. The prepared cytospin slides were stained with Wright–Giemsa staining, and differential cell counts (including macrophages, neutrophils and lymphocytes) were assessed under a light microscope (Olympus BX51, Tokyo, Japan) according to standard morphologic criteria, with a minimum of 400 cells counted per slide. Six samples were evaluated in each group, and all results are presented as means ± SDs.

### 2.6. Histological Examination

Lung tissues were sectioned (5 µm thickness), stained with H.E (hematoxylin and eosin) and observed by a light microscope (Olympus BX51, Tokyo, Japan). A morphological analysis was performed as described previously [31,32] using Image-Pro Plus 6.0 software (Media Cybernetics, Rockville, MD, USA). Three to four visual fields (×100) without tracheae or large blood vessels were randomly selected from each slice. The average alveolar diameter and the density of alveoli were measured and are expressed as the mean linear intercept (MLI) and the mean alveolar number (MAN), respectively. The calculations were as follows: MLI (µm) = total length of the cross/number of alveolar septa lying on the cross; MAN (n/mm^2^) = alveolar number/whole field area. They are usually used to reflect the degree of emphysema. The total bronchial wall area (WAt, total area of bronchus—area of lumen) and the smooth muscle area (WAm, area of outer margin of the smooth muscle—area of medial smooth muscle) were used to evaluate the degree of airway remodeling. They were normalized to Pbm (the basement membrane perimeter) and are presented as WAt/Pbm and WAm/Pbm, respectively. On each slide, three membranous bronchioles were randomly selected for evaluation.

Serial 5 µm sections were stained with H&E, periodic acid–Schiff (PAS) and Masson’s trichrome, respectively. The pathologic changes were scored according to the following parameters: narrowing of the airway lumen, squamous cell metaplasia, goblet cell hyperplasia, inflammatory cell infiltration, connective tissue proliferation, smooth muscle hypertrophy, alveolar structural disturbance and alveolar septal thickening. Each feature was scored using a 5-point scoring system (0, normal; 1, minimal; 2, mild; 3, moderate; and 4, severe), as previously described [29,33], with slight modifications. Scoring results for the different items were combined and expressed as a total score for each individual. Six rats from each group were used for statistical analysis.

### 2.7. TUNEL Analysis

An apoptosis assay of lung tissue was performed using the TUNEL BrightGreen Apoptosis Detection Kit according to the instructions of the manufacturer. Briefly, slides were routinely dewaxed, permeabilized with proteinase K (20 min) and equilibrated with 1 × equilibration buffer (20 min, room temperature), followed by incubation with 50 µL of TUNEL staining solution in the dark (1 h, 37 °C). Subsequently, slides were counterstained with 5 µg/mL DAPI (5 min, room temperature), sealed with glycerol and immediately examined under a fluorescence microscope (Olympus IX73, Tokyo, Japan) at 200 magnification. The TUNEL-positive cells displayed green fluorescence, and blue fluorescence showed the DAPI staining of the nuclei. A total of five fields of view were randomly selected, and the numbers of apoptotic cells and total cells were counted using Image J software (version 1.8.0). The apoptotic index was calculated as the ratio of the number of TUNEL-positive cells to that of total DAPI-stained cells.

### 2.8. Oxidative Stress Assay

The supernatants of lung homogenate were collected, and the levels of MDA, T-AOC and GSH were measured spectrophotometrically using commercial assay kits according to the manufacturer’s instructions. The protein concentration in the samples was determined by the Bradford assay kit. Results were normalized against the total protein contents of samples and are expressed as µmol/mg protein. The ROS in lung tissue was monitored by DCFH-DA based on a previous method [19].

### 2.9. ELISA Assay

The levels of IL-4 and IFN-γ as well as MMP-9 and TIMP-1 in lung tissue were measured at a wavelength of 450 nm using enzyme-linked immunosorbent assay (ELISA) kits according to the manufacturer’s instructions.

### 2.10. Immunohistochemical Analysis

Sections were routinely dewaxed and rehydrated. Then, the sections underwent antigen repair in citrate buffer (0.01 M, pH 6.0) and were incubated with H_2_O_2_ (3%) to eliminate endogenous peroxidase activity. After sealing with 5% BSA, sections were incubated with primary antibodies against Bax (1: 100 dilution), Bcl-2 (1: 300 dilution), Nrf2 (1: 200 dilution), p38 MAPK (1: 200 dilution) and phospho-p38 MAPK (1: 100 dilution) overnight at 4 °C. PBS was used in place of primary antibodies in negative controls. The sections were washed 3 times with PBS and incubated with a suitable secondary antibody (37 °C, 30 min), followed by incubation with SABC (streptavidin–biotin complex) (37 °C, 30 min). The sections were then stained with DAB solution and counterstained with hematoxylin. After sealing with neutral resin, images were observed under a microscope (Olympus BX51, Tokyo, Japan). Brown–yellow particles in the cytoplasm or nucleus were judged as positive. The mean optical density (MOD, MOD = IOD/Area) was evaluated by randomly selecting three non-overlapping fields on a slide using Image-Pro Plus 6.0 software. The results are expressed as mean ± SD from 6 rats per group.

### 2.11. Statistical Analysis

The values are expressed as mean ± standard deviation (SD). SPSS 25.0 software was used for statistical analyses. Normal distribution and homogeneity of variance were checked before use. All data were compared by one-way analysis of variance (ANOVA), followed by the Tukey–Kramer test or Kruskal–Wallis/Dunn’s test. A *p* value of less than 0.05 was considered statistically significant.

## 3. Results

### 3.1. Observation of General Condition

In the control group, the color and luster of the hair were normal. Food and water intake was regular. Rats were active and responsive with stable breathing. During the whole experiment, no deaths were observed. Only at 30 days was there a significant difference in body weight between cigarette smoke (CS) groups. Body weight increased significantly in all groups compared with the initial body weight (*p* < 0.05): 157 g for the control group, 122 g for group 1, 139 g for group 2 and 136 g for group 3; however, the weights of CS groups increased more slowly than that of the control (Figure S2). As the experiment progressed, it was found that physical activity declined, responses were slowed, food and water intake was decreased, and hair became dull and yellow in the “HHY” group, and rats developed cough, dyspnea, sneezing and mental fatigue. However, these symptoms were less severe in the “Zisu” and “Luole” groups. The anatomy showed that the lung tissues of control rats were smooth, shiny, soft, light red in color and normal in size. In CS groups, lung tissues, to varying degrees, lost their luster and became lighter in color, and these effects in the “HHY” group were particularly serious. New-type tobacco may be less irritating to the body than “HHY”.

### 3.2. New-Type Tobacco Caused Less Pathological Damage to Lung Tissues than “HHY”

**Figure 1:**
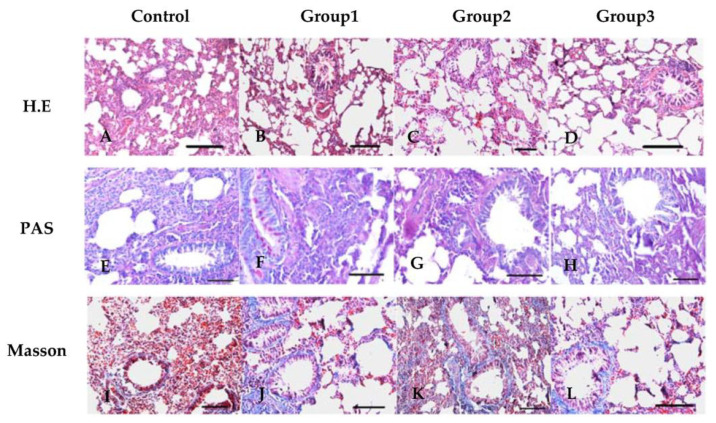

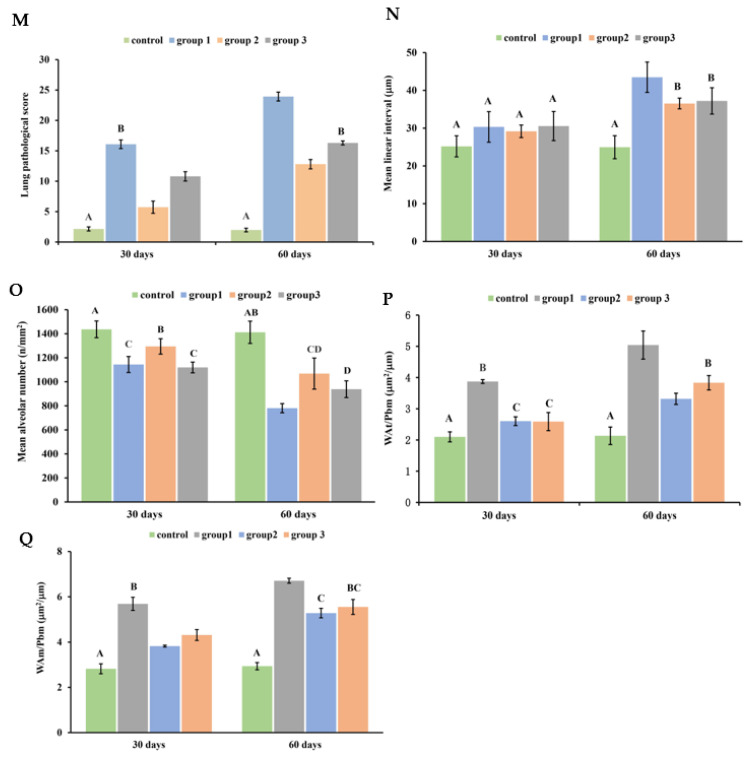
Pathological changes in the lung tissues of cigarette smoke-exposed rats. Representative images of H&E (**A**–**D**), PAS ((**E**–**H**), red-purple indicates mucus production) and Masson’s staining ((**I**–**L**), blue indicates collagen deposition) at 60 days. 200×, scale bars = 50 µm. Lung tissue was observed under a light microscope, and the pathological score was evaluated (**M**). MLI and MAN and WAt and WAm were used as indicators for evaluating emphysema (**N**,**O**) and airway remodeling (**P**,**Q**), respectively. The values are given as means with standard deviations, as shown by the vertical bars (n = 6). The statistical significance between all data is compared. Bars with the same letter(s) indicate no significant differences at the level of *p* < 0.05.

The lung tissues of control rats showed a normal alveolar structure, very little mucus secretion and collagen deposition, and the rare infiltration of inflammatory cells, as shown by H.E, PAS and Masson staining (Figure 1A,E,I and Figure S3A,E,I). CS exposure resulted in histopathological changes to different extents over a period of 60 days, characterized by airway smooth muscle hyperplasia, airway wall thickening, mucus overproduction, infiltration of inflammatory cells into the peribronchiolar and perivascular tissues, excessive collagen deposition, bronchiostenosis and airway obstruction, alveolar enlargement, alveolar septa breakdown, alveolar wall rupture, bullous formation, etc. However, the above changes were more pronounced in the “HHY” group (Figure 1B,F,J and Figure S3B,F,J) than in the “Zisu” (Figure 1C,G,K and Figure S3C,G,K) and “Luole” groups (Figure 1D,H,L and Figure S3D,H,L). The highest pathological scores were seen in the “HHY” group, followed by the “Luole” group and the “Zisu” group at 30 and 60 days (*p* < 0.05) (Figure 1M). Further morphometric analysis showed that there was a significant difference in the indicators of emphysema (MLI and MAN) and airway remodeling (WAt/Pbm and WAm/Pbm) between air- and CS-exposed rats (*p* < 0.05). But compared with the “HHY” group, the values of MLI in the “Zisu” and “Luole” groups were decreased by 16% and 14.4%, and their MAN values were 36.9% and 20.2% higher than those of the “HHY” group, respectively, after 60 days of CS exposure (Figure 1N,O). The levels of WAt/Pbm and WAm/Pbm in the “HHY” group were considerably higher than those in the “Zisu” (51.8% and 27.1%) and “Luole” (31.6% and 20.9%) groups (*p* < 0.05) (Figure 1P,Q). New-type tobacco induced less airway remodeling and emphysematous lesions.

### 3.3. New-Type Tobacco Caused Less Apoptosis in Lung Tissues than “HHY”

**Figure 2:**
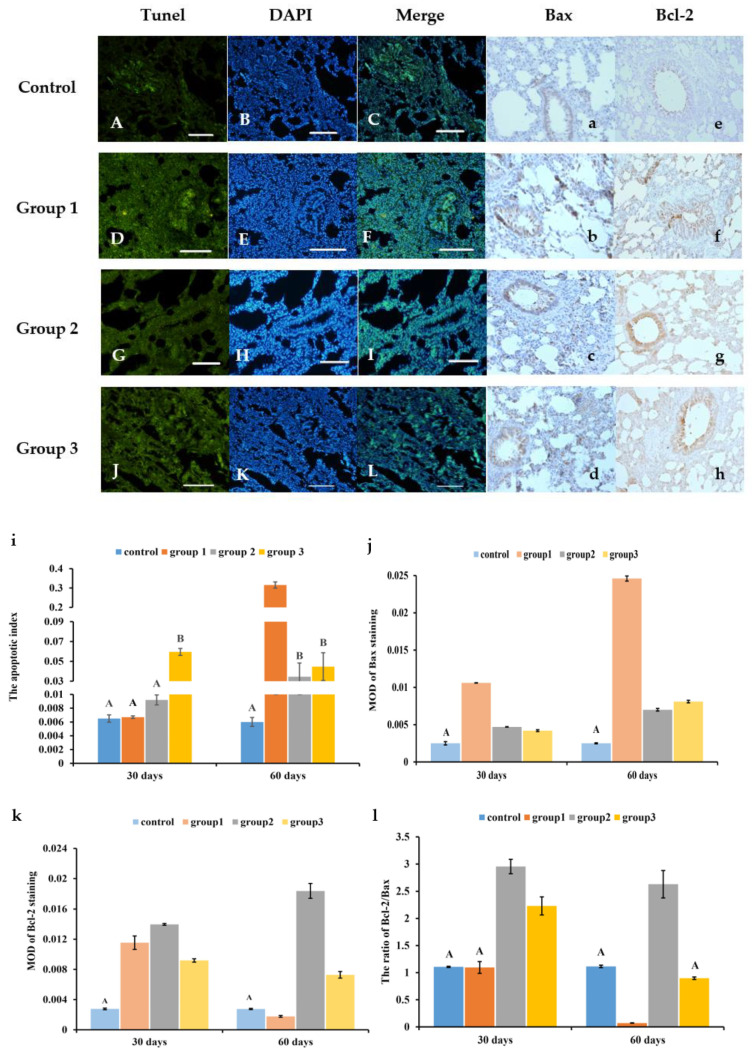
Apoptosis in the lung tissues of cigarette smoke-exposed rats. Apoptosis in lung tissue was evaluated by the TUNEL assay (**A**–**L**), followed by the calculation of the apoptosis index (**i**). Immunohistochemical staining of Bax (**a**–**d**) and Bcl-2 (**e**–**h**) protein. Representative images at 60 days. 200×, scale bars = 50 µm. The relative expression levels of Bax (**j**) and Bcl-2 (**k**). The ratio of Bcl-2 to Bax (**l**). The data are presented as the means with standard deviations, as shown by the vertical bars (n = 6). The statistical significance between all data is compared. Bars with the same letter indicate no significant differences at the level of *p* < 0.05.

As shown in Figure 2A–L and Figure S4A–L, the number of TUNEL-positive cells in all CS exposure groups was higher than that in the control group. CS greatly increased the apoptotic index (AI) and promoted the widespread expression of Bax protein in lung tissues as compared with the control. But at 30 days and 60 days, the expression of Bcl-2 in the “Zisu” group showed different changes compared with the “HHY” and “Luole” groups (Figure 2a–h and Figure S4a–h). The AI of the “Zisu” and “Luole” groups was reduced by 89.1% and 85.8% compared with that of the “HHY” group, respectively, after 60 days of exposure (Figure 2i). The relative expression level of Bax in the “HHY” group was 2.5 and 2.0 times higher than that in the “Zisu” and “Luole” groups, but its Bcl-2 expression level was 90.3% and 75.6% lower than that in the “Zisu” and “Luole” groups (Figure 2j,k), which resulted in a higher ratio of Bcl-2 to Bax in the “Zisu” and “Luole” groups than in the “HHY” group (*p* < 0.05), especially in the “Zisu” group (Figure 2l).

### 3.4. New-Type Tobacco Caused Less of an Increase in the Numbers of Total Leukocytes and Neutrophils in the BALF than “HHY”

**Figure 3:**
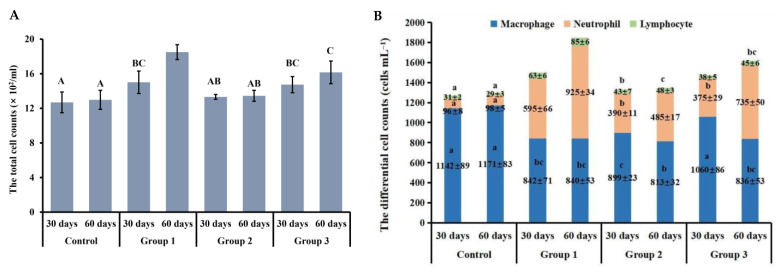
Leukocytosis and neutrophilia in the BALF of cigarette smoke-exposed rats. (**A**) The total number of inflammatory cells. (**B**) The differential cell counts. Results are presented as means with standard deviations, as shown by the vertical bars (n = 6). The statistical significance between all data is compared. Bars with the same uppercase or lowercase letters(s) indicate no significant differences at the level of *p* < 0.05.

Cigarette smoke increased the recruitment of inflammatory cells, especially neutrophils, into the BALF as compared with air exposure. At 30 days, there were no obvious significant differences in the total number of leukocytes between CS groups (*p* > 0.05), but the number in the “HHY” group was 37.5% and 14.5% higher than those in the “Zisu” and “Luole” groups, respectively, at 60 days (*p* < 0.05) (Figure 3A). The numbers of macrophages, neutrophils and lymphocytes in the BALF also showed significant changes. Compared with the control, there was a substantial decrease in the macrophage number and an increase in the neutrophil number in smoke-exposed rats at 30 days and 60 days (*p* < 0.05), which may be due to the migration of macrophages into the lung interstitium and the massive influx of neutrophils into the BALF. Although lymphocytes accounted for the smallest proportion, their number also markedly increased. The neutrophil number in the “Zisu” and “Luole” groups was 34.5% and 37% lower at 30 days and 47.6% and 20.5% lower at 60 days than that in the “HHY” group, respectively (Figure 3B).

### 3.5. New-Type Tobacco Caused Less Oxidative Stress in Lung Tissues than “HHY”

**Figure 4:**
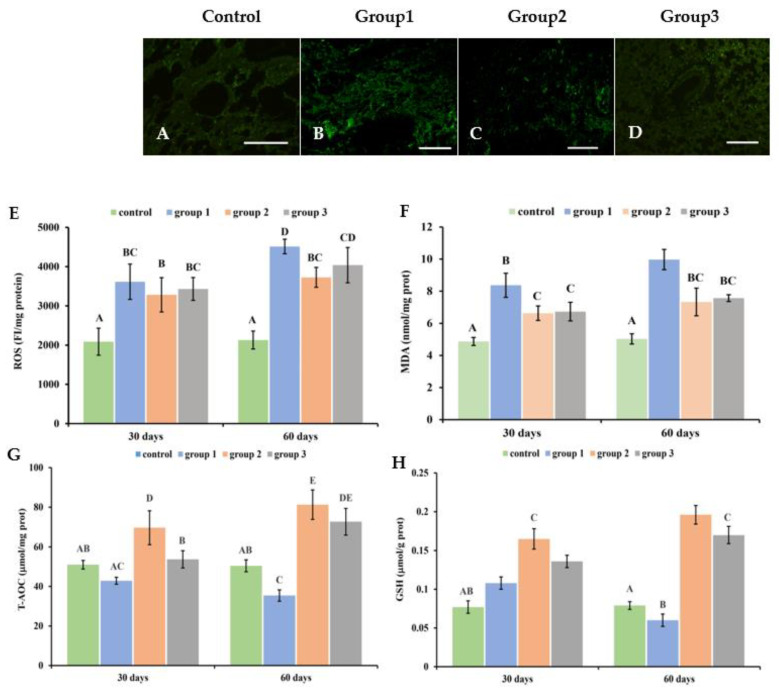
Oxidative stress in the lung tissues of cigarette smoke-exposed rats. DCFH-DA staining of ROS (**A**–**D**). Representative images at 60 days. 200×, scale bars = 50 µm. (**E**) Contents of reactive oxygen species (ROS). (**F**) Malondialdehyde (MDA) levels. (**G**) Total antioxidant capacity (T-AOC). (**H**) Reduced glutathione (GSH) levels. The values are given as means with standard deviations, as shown by the vertical bars (n = 6). The statistical significance between all data is compared. Bars with the same letter(s) indicate no significant differences at the level of *p* < 0.05.

Cigarette smoke induces oxidative stresses in lung tissues through the overproduction of ROS, causing the elevation of MDA levels and the depletion of antioxidant defenses. This effect was most pronounced in the “HHY” group. In CS groups, stronger green fluorescence was observed in lung tissue when compared with the control group (Figure 4A–D and Figure S3M–P), and ROS levels progressively increased in a time-dependent manner (*p* < 0.05) (Figure 4E). The ROS level in the “HHY” group was 21.1% and 11.8% higher than that in the “Zisu” and “Luole” groups after 60 days of exposure. The MDA content in the “Zisu” and “Luole” groups decreased by 26.5% and 24.1%, respectively (Figure 4F). However, the levels of T-AOC and GSH were markedly decreased in the “HHY” group. But, interestingly, these levels were greatly enhanced in the “Zisu” and “Luole” groups (*p* < 0.05). The levels of T-AOC and GSH in the “Zisu” and “Luole” groups were more than 1 and 2 times higher than those in the “HHY” group (Figure 4G,H). There were no significant differences in ROS, MDA and T-AOC levels between the “Zisu” and “Luole” groups (*p* > 0.05).

### 3.6. New-Type Tobacco Altered the Ratios of MMP-9/TIMP-1 and IFN-γ/IL-4 in Lung Tissue to a Lesser Degree than “HHY”

**Figure 5:**
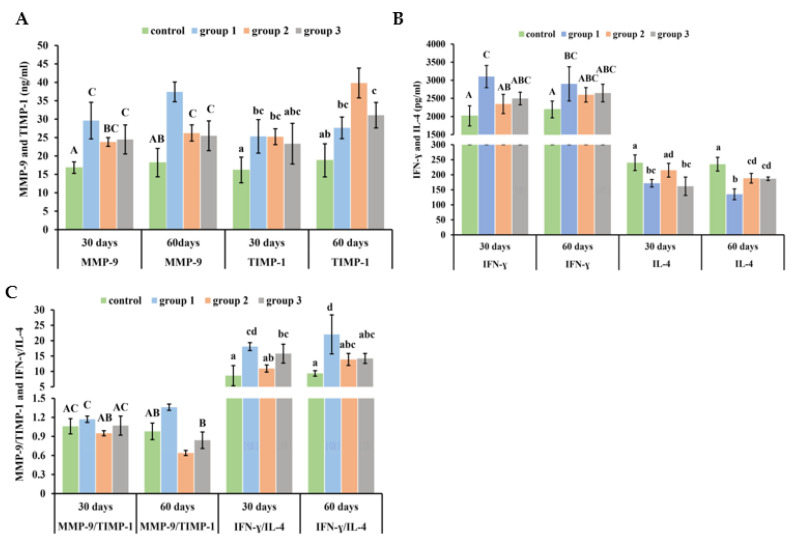
The imbalance of MMP-9/TIMP-1 and IFN-γ/IL-4 in the lung tissues of cigarette smoke-exposed rats. The levels of MMP-9 and TIMP-1 (**A**). The contents of IFN-γ and IL-4 (**B**). The ratios of MMP-9/TIMP-1 and IFN-γ/IL-4 (**C**). Results are presented as means with standard deviations, as shown by the vertical bars (n = 6). The statistical significance between all data is compared. Bars with the same uppercase or lowercase letters(s) indicate no significant differences at the level of *p* < 0.05.

Rats exposed to CS presented higher levels of MMP-9 and TIMP-1 in lung tissues compared with control rats. At 30 days, there were no significant differences between CS groups. At 60 days, the MMP-9 concentration in the “Zisu” and “Luole” groups was reduced by 30% and 31.9%, but their TIMP-1 concentrations increased by 44.1% and 12.4%, respectively, when compared to the “HHY” group (Figure 5A). However, CS increased IFN-γ but decreased IL-4 levels. At 30 days, only the “HHY” and “Zisu” groups showed obvious differences. At 60 days, IL-4 levels in the “HHY” group were significantly lower when compared with the other three groups (*p* < 0.05) (Figure 5B). The effect of CS on the protease/antiprotease imbalance and Th1/Th2 immune imbalance was usually indicated by the ratios of MMP-9/TIMP-1 and IFN-γ/IL-4. The ratio of MMP-9 to TIMP-1 was higher in the “HHY” group than in the control group, but it was lower in the “Zisu” and “Luole” groups than in the control group. In contrast, CS could increase the ratio of IFN-γ to IL-4. But the ratio of IFN-γ to IL-4 in the “Zisu” and “Luole” groups was lower than that in the “HHY” group (*p* < 0.05) (Figure 5C).

### 3.7. New-Type Tobacco Altered the Expression of Nrf2 and p38MAPK

**Figure 6:**
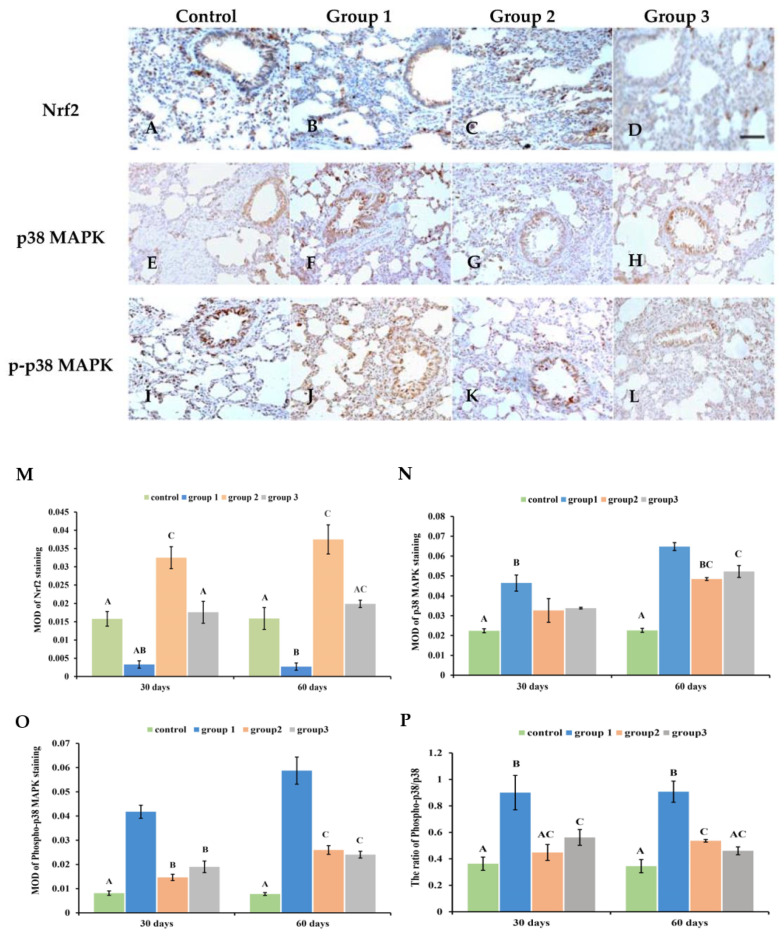
Cigarette smoke-altered Nrf2 and p38 MAPK expression in the lung tissues of rats. Immunohistochemical staining of Nrf2 (**A**–**D**), p38 MAPK (**E**–**H**) and phospho-p38MAPK (**I**–**L**). Representative images at 60 days. 200×, scale bars = 50 µm. The relative expression levels of Nrf2 (**M**), p38 MAPK (**N**) and phosphor-p38 MAPK (**O**). The ratio of phosphor-p38 to p38 (**P**). Results are presented as means with standard deviations, as shown by the vertical bars (n = 6). The statistical significance between all data is compared. Bars with the same letter(s) indicate no significant differences at the level of *p* < 0.05.

The distribution and expression of Nrf2 and p38 MAPK were analyzed using immunohistochemical staining. It was found that there was a small amount of positive brown staining in the control group (Figure 6A,E,I and Figure S5A,E,I). In the “HHY” group, less staining of the Nrf2 protein was seen, but p38MAPK and its phosphorylation were widely expressed in pulmonary tissues (Figure 6B,F,J and Figure S5B,F,J). After “Zisu” and “Luole” cigarette exposure, it was found that the Nrf2 protein was widely distributed in the bronchi, alveoli and lung interstitium (Figure 6C,D and Figure S5C,D). The positive staining of p38MAPK and p-p38MAPK in these parts was significantly decreased compared with those exposed to the “HHY” cigarette (Figure 6G,H,K,L and Figure S5G,H,K,L). A quantitative analysis showed that the three cigarettes had significantly different effects on the expression levels of total Nrf2 when compared with the control group (*p* < 0.05) (Figure 6M). All CS exposure markedly improved the expression level of p38MAPK and p-p38MAPK (*p* < 0.05). But the level of p38MAPK in the “HHY” group was 33.9% and 24.1% higher than those in the “Zisu” and “Luole” groups. The level of p-p38MAPK in the “Zisu” and “Luole” groups decreased by 55.8% and 59.2% compared with the “HHY” group (Figure 6N,O). It was further found that the three cigarettes altered the ratio of p-p38MAPK/p38MAPK to different degrees. The ratio in the “HHY” group was 69.2% and 96.7% higher than in the “Zisu” and “Luole” groups (Figure 6P). The Nrf2 and p38MAPK pathways may play an important part in CS-induced oxidative stress, cell apoptosis, the inflammatory response and pathological damage.

## 4. Discussion

At present, of the over 5000 identified constituents of cigarette smoke, about 150 are considered “tobacco smoke toxicants”. It is estimated that there are about 1 × 10^16^ free radicals per cigarette, or 5 × 10^14^ per puff [34,35]. Although the specific constituents responsible for COPD have not been identified, oxidant stress, which is caused by exogenous oxidants in the inhaled CS and endogenous ROS from the activation of inflammatory cells, is regarded as the central factor in the pathogenesis of COPD [3,36]. Some medicinal and aromatic plants have been used in cigarettes to remove or reduce free radicals and other harmful components of smoke or to manufacture a “less hazardous” “herbal” cigarette [5,7,37]. However, some scholars believed that this could instead enhance smoke toxicity because they could undergo intricate reactions during smoking, forming “new” constituents or increasing the concentrations of existing constituents. For example, some volatile components may be directly transferred to cigarette smoke, while other nonvolatile ones may undergo combustion and pyrolysis [10,22,38].

The tobacco type, or even the variety, is an important determining factor for the chemical composition of cigarette smoke [9,11,12]. Flue-cured tobacco is characterized by large amounts of carbohydrates, such as sugars and starch, but burley tobacco has more nitrogen components, such as amino acids and nitrate. Because they may be the main precursors of certain smoke constituents, the yield of nitrogenous toxicants is higher in the smoke of burley tobacco, but formaldehyde and catechol are higher in flue-cured tobacco smoke. And flue-cured tobacco exhibited greater toxicity than burley tobacco [34,39,40]. Our previous study indicated that the contents of total sugars, reducing sugars and nicotine in “Luole” were higher than those in “HHY”, but the contents of total nitrogen and protein were lower. The contents of four conventional chemical substances, namely, protein, total nitrogen, sugars and alkaloids, were higher in “Zisu” compared with “HHY”. Although only over 100 compounds have been identified to date in new-type tobacco varieties, it has been found that there are as many as 50 different substances between new-type tobacco and “HHY”, as well as “Zisu” and “Luole” [17,18,19]. Some secondary metabolites, such as terpenoids, have been demonstrated to attenuate oxidative stress, inhibit apoptosis and regulate inflammatory responses in airway and lung epithelial cells, which might be less harmful to pulmonary tissue stability and functions [41]. In vitro assays such as the Ames assay and neutral red uptake assay have been recommended by CORESTA (Co-operation Centre for Scientific Research Relative to Tobacco) to evaluate and compare the mutagenicity and cytotoxicity of cigarette smoke. New-type tobacco was found to be significantly less cytotoxic and mutagenic than “HHY” [18,42,43].

Usually, in vitro tests are performed with cigarette smoke extract (CSE), which might lose volatiles and some reactive components (such as particulate components) due to filtration. So, it does not fully reflect the toxic effects of cigarette smoke. An in vivo study, namely, on specific cigarette smoke-induced diseases such as COPD, was recommended for further evaluation [10,26]. Animal models of COPD have mainly been used to study its pathogenesis or evaluate the preventive and treatment effects of medication, including medicinal plant extracts and active constituents, for a long time [23,44,45]. Rat COPD models have been constructed for a variety of cigarette types and exposure methods so far. “Nose-only” or “whole-body” exposure has been a common method for delivering cigarette smoke to animals [28,29,30]. Two kinds of smoke are generated during smoking, i.e., mainstream smoke (MS), inhaled directly by the smoker, and sidestream smoke (SS), emitted from the burning end of a cigarette. SS is regarded as more hazardous than MS, because it contains more harmful compounds than MS. The discrepancy is strongly correlated with the different combustion conditions during the process of puffing and smoldering [9,24,46,47]. In this study, all test cigarettes were made only from pure leaves to ensure that smoke chemicals were produced by a single tobacco type. Subsequently, all animals were subjected to whole-body exposure for two hours per day for 6 days per week and for a minimum of 60 days. Smoking rats could be induced to show obvious COPD-like pathological hallmarks, such as chronic inflammation, airway remodeling and emphysema. But no new histopathological alterations were observed in the “Zisu” and “Luole” exposure groups. A quantitative analysis further showed decreased MAN as well as increased MLI, WAt and WAm, suggesting that the rat COPD model was successfully established [48].

*Perilla frutescens* and *Ocimum basilicum*, aromatic plants in the Lamiaceae family, are also identified as medicinal and edible plants with high activity, low toxicity and high safety. They have been cultivated since ancient times and have been widely used in traditional medicine for respiratory diseases such as influenza, cough, asthma and chronic bronchitis in China. It has been known that *P. frutescens* has antipyretic, spasmolytic, antiasthmatic, antitussive, expectorant and restorative properties. The extract of *P. frutescens* could significantly inhibit ROS production by neutrophils. Members of the genus *Ocimum* have been demonstrated to have a wide range of biological activities, such as antioxidant, anti-inflammatory and immunity-modulating properties [49,50,51]. These characteristics make them perfect genetic resources for tobacco improvement.

In the lung, cigarette smoke places 50 different types of cells under oxidative stress, triggering inflammation, protease/antiprotease imbalances and apoptosis, which are responsible for the development of COPD [3,30,44,52]. Generally, enzymatic and non-enzymatic antioxidant systems, such as SOD, CAT and GSH, protect cells from an oxidative challenge, among which GSH depletion is thought to be a key step in the toxic effects of smoke [40]. ROS not only disrupt the cellular oxidation/antioxidation balance but also result in oxidative injury to macromolecules. MDA levels are often used as a biomarker of oxidative damage. In the lung tissue, cigarette smoke induces the recruitment and activation of inflammatory cells, which release ROS and proteolytic enzymes such as matrix metalloproteinases (MMPs) [53]. If not sufficiently counterbalanced by antiproteases (the tissue inhibitors of MMP, TIMPs) and antioxidant factors, they cause the development of emphysema. An imbalanced MMP-9/TIMP-1 ratio is an important determinant in the pathogenesis of emphysema [54].

This study also found that there were significant increases in the total leukocyte count and neutrophil number in the BALF. Neutrophils are major contributors to lung injury because they can release a lot of cytotoxic mediators, like cytokines, chemokines, proteases and oxygen-derived free radicals [55,56,57]. New-type tobacco could cause less of an increase in neutrophils and infiltration in lung tissue. There is a good balance between Th1 and Th2 immune responses in healthy individuals. But COPD is mainly characterized by the Th1 type, and a Th1/Th2 imbalance (i.e., IFN-γ/IL-4) is a key factor leading to airway inflammation and remodeling in COPD [4,50].

Apoptosis of structural cells is one of the important mechanisms of COPD [4,58]. Apoptosis, as evaluated by TUNEL staining, greatly increased in the bronchial epithelium, bronchial wall, alveolar epithelial and alveolar septum of rats exposed to cigarette smoke. Even without the accumulation of inflammatory cells, apoptosis is sufficient to cause emphysema in some COPD models. Meanwhile, Bax protein was mainly expressed in alveolar epithelial cells of patients with emphysema, but not in healthy individuals. The proapoptotic functions of Bax can be antagonized by antiapoptotic proteins like Bcl-2. The upregulation of antiapoptotic activity may potentially protect the bronchial epithelium and prevent emphysema development in mice exposed to cigarette smoke [58].

Studies have shown that the crosstalk between MAPK and Nrf2 signaling pathways is closely related to multiple pathological processes of COPD, such as oxidative stress, airway inflammation, mucus hypersecretion and epithelial cell apoptosis [3,6]. In particular, the activation of p38 MAPK regulates the expression of various inflammatory mediators, such as IL-8, TNF-α and MMPs, which contribute to the recruitment and activation of neutrophils [19,25]. Nrf2, a redox-sensitive transcription factor, plays an important role in protecting the lungs from CS-induced oxidative injury, immune responses and apoptosis by upregulating nearly 50 genes involved in antioxidant activity, cytoprotection and detoxification [56,59]. It has been found that there was a significant decrease in Nrf2 protein levels in COPD lungs [60]. Nrf2-deficient mice showed an increased susceptibility to CS-induced lung damage [61]. If this protection fails, the escalation of the oxidative burden could result in p38 MAPK activation.

## 5. Conclusions

This study indicated that new-type tobacco induced less oxidative stress and apoptosis, lessened the increase in total leukocyte counts and neutrophil numbers and altered the ratios of MMP-9/TIMP-1 and IFN-γ/IL-4, as well as the expression of Nrf2 and p38 MAPK, in rat lungs compared with “HHY”, causing less damage to the airways and lung parenchyma. In essence, the differences likely resulted from differences in the smoke components of different tobacco. Based on previous in vitro and the present in vivo studies, new-type tobacco may be less hazardous than regular tobacco. But it may not yet be a truly “safer” alternative to regular tobacco, and its effects on health still need long-term observation. The majority of toxic smoke substances are formed by pyro-synthesis from the components of tobacco leaves. Introducing genes from some medicinal plants via distant hybridization could be an attractive strategy for tobacco harm reduction.

## Data Availability

The data presented in this study are available upon request from the corresponding author.

## Supplementary Materials

The following supporting information can be downloaded at https://www.mdpi.com/article/10.3390/antiox13050511/s1: Figure S1: The breeding materials; Figure S2: Body weight changes in CS groups and normal group; Figure S3: Pathologic alterations in lung tissues of CS-exposed rats at 30 days; Figure S4: Apoptosis in lung tissues of CS-exposed rats at 30 days; Figure S5: Cigarette smoke-altered Nrf2 and p38 MAPK expression in lung tissues of rats at 30 days.
