# Supplementary material for: Differences in Airway Remodeling and Emphysematous Lesions between Rats Exposed to Smoke from New-Type and Conventional Tobacco Varieties
Source: Antioxidants (Basel). 2024 Apr 24;13(5):511. doi: 10.3390/antiox13050511 (PMC11117731; doi:10.3390/antiox13050511)
Supplement: Supplementary file 1 [file antioxidants-13-00511-s001.zip › antioxidants-2941119-supplementary (1).pdf]

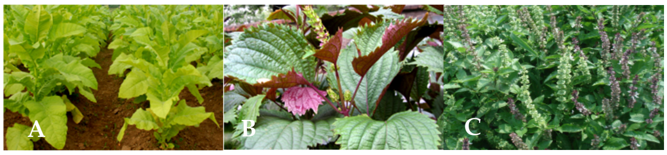

Supplemental Figure S1. The breeding materials. (**A**) *Nicotiana tabacum* L., (**B**) *Perilla frutescens* var. *Frutescens* and (**C**) *Ocimum basilicum* L.

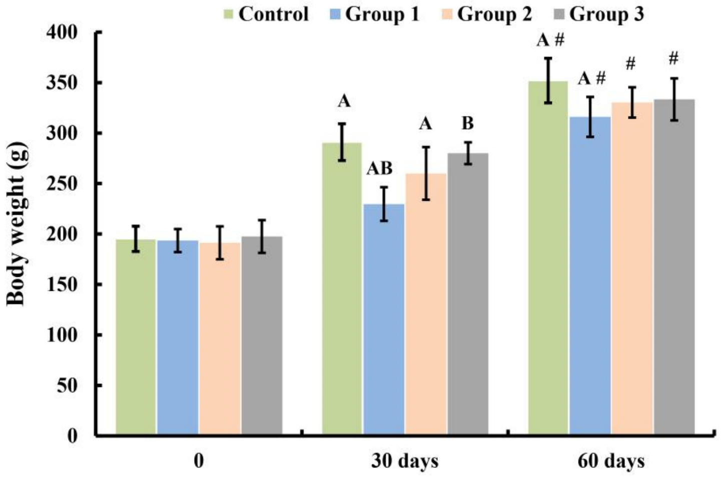

Supplemental Figure S2. Body weight changes in CS groups and normal groups. The same uppercase letter(s) on the bars indicated significant differences between groups at 30 days or 60 days, respectively (P<0.05). # indicated significant differences in each group at 60 days compared to the initial body weight (P<0.05).

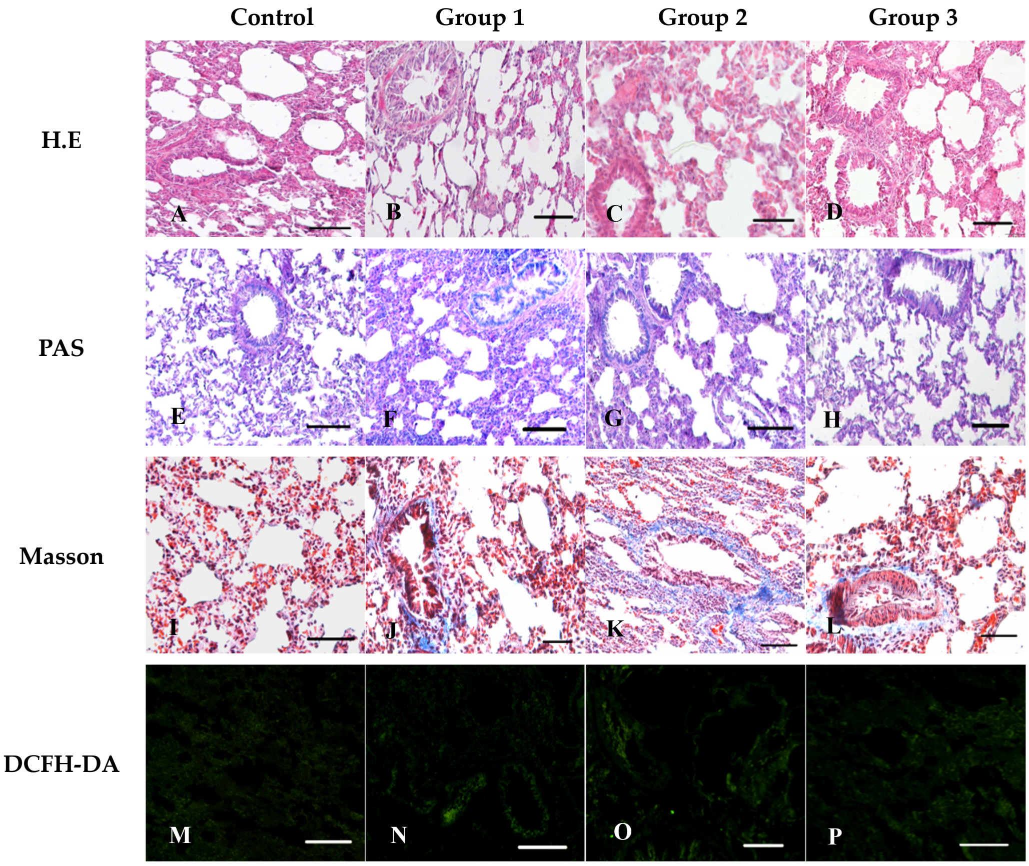

Supplemental Figure S3. Pathological changes in lung tissues of CS-exposed rats at 30 days. Representative images of H&E (**A**-**D**), PAS (**E**-**H**), Masson' s (**I**-**L)** and DCFH-DA (**M**-**P**) staining.

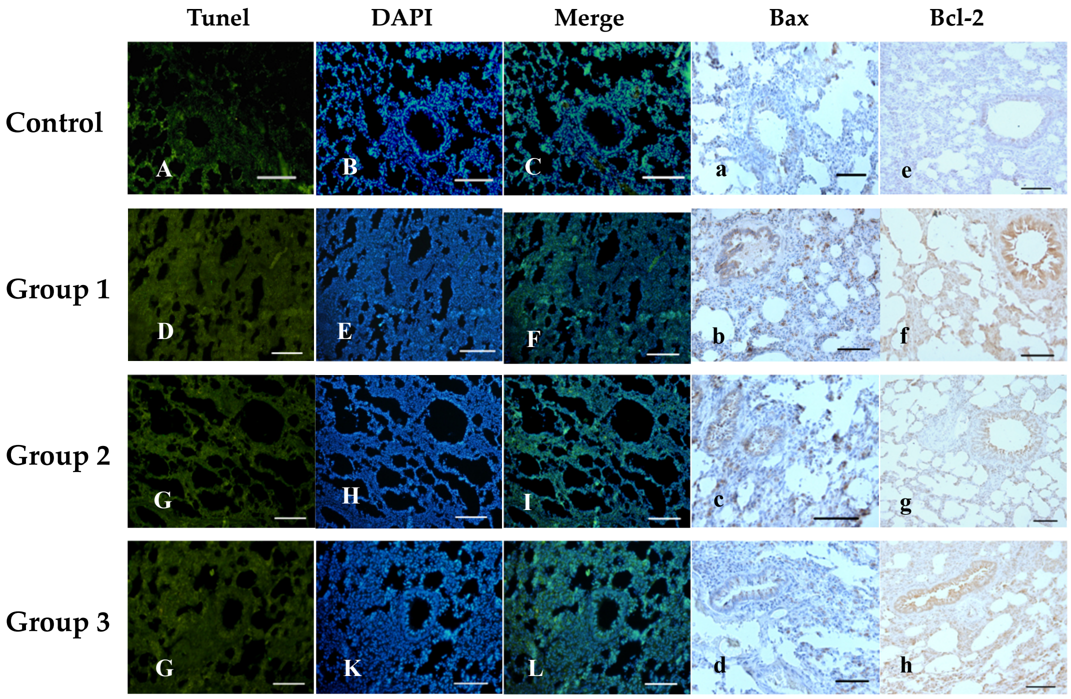

Supplemental Figure S4. Apoptosis in lung tissues of CS-exposed rats at 30 days. Apoptosis of lung tissue was evaluated by TUNEL assay (**A**-**L**)**.** Representative images of immunohistochemical staining of Bax (**a-d**) and Bcl-2 (**e-h**) protein.

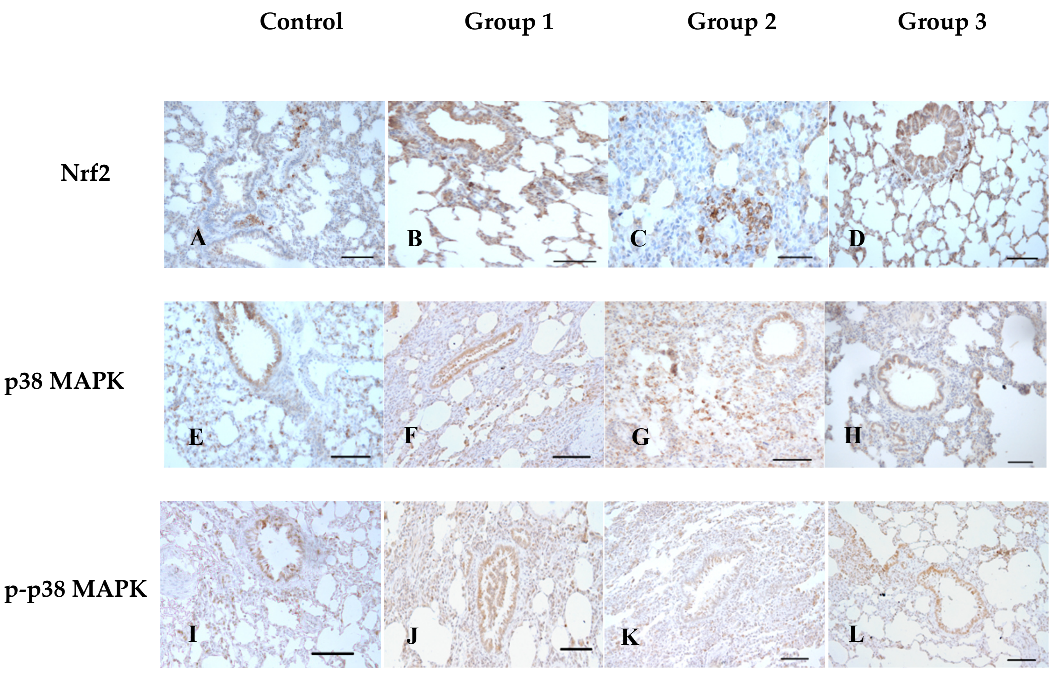

Supplemental Figure S5. Cigarette smoke altered Nrf2 and p38 MAPK expression in lung tissues of rats at 30 days. Representative images of immunohistochemical staining of Nrf2 (**A-D**), p38 MAPK (**E-H**) and phospho-p38MAPK (**I-L**).
